# Smartphones as an Ecological Niche of Microorganisms: Microbial Activities, Assembly, and Opportunistic Pathogens

**DOI:** 10.1128/spectrum.01508-22

**Published:** 2022-08-30

**Authors:** Jintao He, Xiaoqiang Shen, Nan Zhang, Chao Sun, Yongqi Shao

**Affiliations:** a Max Planck Partner Group, Faculty of Agriculture, Life and Environmental Sciences, Zhejiang University, Hangzhou, China; b Analysis Center of Agrobiology and Environmental Sciences, Zhejiang University, Hangzhou, China; c Key Laboratory for Molecular Animal Nutrition, Ministry of Education, Beijing, China; Temasek Life Sciences Laboratory

**Keywords:** smartphone microorganism, microbial community assembly, skin microbiota, smartphone hygiene, human health, potential pathogen, surface microbiota

## Abstract

Smartphone usage and contact frequency are unprecedentedly high in this era, and they affect humans mentally and physically. However, the characteristics of the microorganisms associated with smartphones and smartphone hygiene habits remain unclear. In this study, using various culture-independent techniques, including high-throughput sequencing, real-time quantitative PCR (RT-qPCR), the ATP bioluminescence system, and electron microscopy, we investigated the structure, assembly, quantity, and dynamic metabolic activity of the bacterial community on smartphone surfaces and the user’s dominant and nondominant hands. We found that smartphone microbiotas are more similar to the nondominant hand microbiotas than the dominant hand microbiotas and show significantly decreased phylogenetic diversity and stronger deterministic processes than the hand microbiota. Significant interindividual microbiota differences were observed, contributing to an average owner identification accuracy of 70.6% using smartphone microbiota. Furthermore, it is estimated that approximately 1.75 × 10^6^ bacteria (2.24 × 10^4^/cm^2^) exist on the touchscreen of a single smartphone, and microbial activities remain stable for at least 48 h. Scanning electron microscopy detected large fragments harboring microorganisms, suggesting that smartphone microbiotas live on the secreta or other substances, e.g., human cell debris and food debris. Fortunately, simple smartphone cleaning/hygiene could significantly reduce the bacterial load. Taken together, our results demonstrate that smartphone surfaces not only are a reservoir of microbes but also provide an ecological niche in which microbiotas, particularly opportunistic pathogens, can survive, be active, and even grow.

**IMPORTANCE** Currently, people spend an average of 4.2 h per day on their smartphones. Due to the COVID-19 pandemic, this figure may still be increasing. The high frequency of smartphone usage may allow microbes, particularly pathogens, to attach to—and even survive on—phone surfaces, potentially causing adverse effects on humans. We employed various culture-independent techniques in this study to evaluate the microbiological features and hygiene of smartphones, including community assembly, bacterial load, and activity. Our data showed that deterministic processes drive smartphone microbiota assembly and that approximately 1.75 × 10^6^ bacteria exist on a single smartphone touchscreen, with activities being stable for at least 48 h. Fortunately, simple smartphone cleaning/hygiene could significantly reduce the bacterial load. This work expands our understanding of the microbial ecology of smartphone surfaces and might facilitate the development of electronic device cleaning/hygiene guidelines to support public health.

## INTRODUCTION

Smartphones are now one of the most commonly used personal items. Global smartphone users increased by 40% from 2016 to 2020, reaching 3.5 billion, and these users spent an average of 4.2 h per day on their phones (1). Due to the COVID-19 pandemic, these figures may still be increasing (2, 3). Currently, the effects of smartphones on people’s mental and physical well-being manifest in various ways (4, 5). It has been shown that, on average, people touch their phones 2,617 times per day (6); this number may be even higher in the current era. Despite recent studies having focused on problems associated with smartphones (e.g., sleep disturbance, mental issues, and nonionizing radiation) (7–9), microbial features and smartphone hygiene are largely ignored aspects of research (the small amount of research that has been done has mostly involved hospital workers) (10).

The hand represents a critical connection between humans and the external world and plays a vital role as a vector by which microorganisms are transmitted among body sites, individuals, items, and the environment (11). The hand microbiota could thus contribute to the formation of a microbial fingerprint on our possessions (12). Since smartphones are one of the items we contact with high frequency, it is not surprising that the features of microbial communities on smartphones could present high similarities with those of the owners’ hands (13, 14) with the potential even for identification for forensic purposes (15). A previous study found personalized traits and similarities in microbiome composition between the thumb, index finger, and smartphone (13). Nevertheless, there is still a limited understanding of the relationship between the hand (the dominant and nondominant hands [DH and NH, respectively]) and smartphones. Furthermore, little is known about the assembly processes of microbial communities on smartphones. Are smartphone microbiotas transferred from human hands randomly? Is their assemblage governed by deterministic processes in the smartphone microenvironment? Do transferred microorganisms form a microbial community and interact with each other?

Moreover, extensive and active pathogen contamination was reported for the surfaces of the phones of university students (10, 16) and hospital workers, including intensive care unit (ICU) workers (17, 18). The existence of characteristic and perhaps active microbial communities on smartphones indicates the potential of these items as a reservoir of human pathogens. Furthermore, people attach little importance to phone hygiene. A recent report showed that most of the studied clinicians disinfected their phones only when they were dirty (18). Therefore, smartphones, as a pathogen reservoir, might undermine our sanitation efforts.

To date, research on smartphone-associated microorganisms has involved mostly culture-dependent methods (17–19). In this study, we investigated the microbial communities on the hands of 14 healthy volunteers and the surfaces of their smartphones. Several culture-independent techniques were employed to characterize the microbial communities, including high-throughput sequencing, real-time quantitative PCR (RT-qPCR), the ATP bioluminescence system, and electron microscopy. In particular, the ATP bioluminescence system is widely used to monitor hygiene practices in food industries and the health care environment (20). The aims of the present study were to (i) evaluate the composition and relationship of microbiotas on smartphones, dominant hands, and nondominant hands, (ii) elucidate the assembly processes (determinism or stochasticity) of smartphone microbiotas, (iii) assess the potential of the smartphone microbiome profile for identifying the owner, and (iv) quantify the absolute abundance and activity of bacteria on smartphones. We hypothesized that smartphone microbiotas primarily originate from human hands, given the high touch frequency of smartphones in this era. Thus, smartphone microorganisms were expected to be in a state of rapid species turnover (transient), and thus, there is no sufficient time, resources, or activity to form a “community” and interact with each other, contributing to the same or more stochastic assembly processes among smartphone microbiotas as among hand microbiotas.

## RESULTS

### Characterization of bacterial communities on the smartphone surface (SS) and the owner’s dominant and nondominant hands (DH and NH).

**FIG 1:**
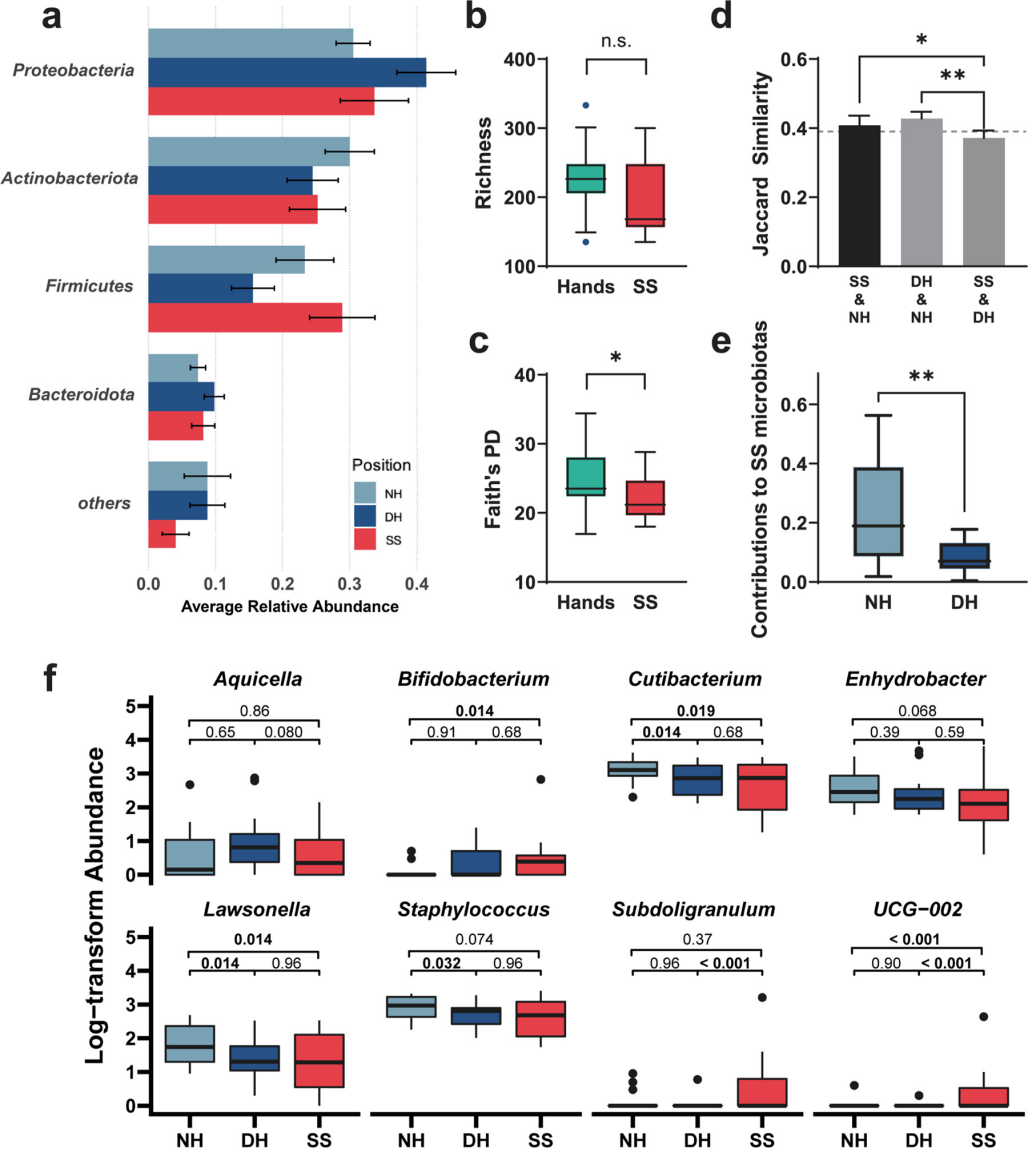
Smartphone microbiotas have lower phylogenetic diversity than hand microbiotas and are more similar to nondominant hand (NH) microbiotas than to dominant hand (DH) microbiotas. (a) Bar plot showing the four most abundant bacterial phyla represented on the NH, DH, and smartphone surface (SS). No significant difference was observed between the two hands and SS (Kruskal-Wallis H test). (b and c) Tukey boxplot of alpha diversity of the communities on hands and SS measured by the richness index (b) and Faith’s phylogenetic diversity (PD) (c) index. Dots represent outliers. (d) Bar chart representing three sets of beta diversity, based on Jaccard similarity, between each owner’s NH, DH, and SS. The error bar represents the standard error. (e) The potential contribution of NH and DH microbiotas to the SS microbiota in each individual, based on source tracking analysis using FEAST. (f) Abundances of significantly changed genera in each group based on DESeq2. False-discovery rate (FDR)-corrected *P* values are shown. Box plots show median (line), 25th and 75th percentiles (box), and 1.5× the interquartile range (IQR, whiskers). Dots represent outliers. *, *P *≤ 0.05; **, *P *≤ 0.01. n.s., nonsignificant  (*P > *0.05).

After quality control and sample pairing, the bacterial communities of 14 graduate students were characterized using amplicon sequencing. Detailed information on the subjects with their anonymous serial numbers (V1 to V14) is available in the supplemental material (Table S1). After sequence processing and rarefaction, 1,062 unique bacterial operational taxonomic units (OTUs) were identified. According to the taxonomic classification, the bacteria belonged mainly to *Proteobacteria*, *Actinobacteria*, *Firmicutes*, and *Bacteroidetes* (Fig. 1a), accounting for a mean relative abundance of 92.7%. Here, the OTUs present in all the samples (both hand and smartphone) were defined as the core OTUs. The taxonomy and average abundance of all 16 core OTUs, including Cutibacterium acnes, Staphylococcus, Streptococcus, Moraxella osloensis, are displayed in Table S2. The core OTUs accounted for an average relative abundance of 48.56% in each sample.

To further reveal the differences between hands and SS, the alpha diversity of each community was calculated (Table S3). Smartphone microbiotas are expected to originate from hand microbiotas and be transient and, therefore, to have comparable diversity. No significant difference was observed between hands and SS based on the Chao1 and Sobs richness indices (*P = *0.165 and 0.110, respectively, *t* test [Fig. 1b and Table S3]), neither of which incorporated the phylogenetic information among taxa. In contrast, unexpectedly, Faith’s phylogenetic diversity (PD) (Fig. 1c) of the bacterial communities on SS was significantly lower than that on hands (*P = *0.038, *t* test). Additionally, phylogeny-based metrics (weighted UniFrac dissimilarities, 29.61% and 18.23%) could better explain the variation in the microbiota composition than taxonomy-based metrics (Bray-Curtis dissimilarities, 18.83% and 13.08%) in the first two axes of the multidimensional scaling, confirming the altered phylogenetic structure of the microbiota on the SS. However, no major variation was observed between hands and SS based only on difference analyses (ADONIS, *R*^2^ = 0.024 and *P = *0.449; analysis of similarity [ANOSIM], *R *= 0.136 and *P = *0.046) (Table 1 and Table S4). Given that the difference between hands and SS might be masked by similarity *per se* or high interindividual difference (Table 1 and Table S5), we further dissected the masking effects of interindividual variation by considering the interaction effect using two-way ADONIS (Table 2) and found significant differences between hands and SS (hands/SS, *R*^2^ = 0.027 and *P = *0.025; individual, *R*^2^ = 0.572 and *P < *0.001; interaction effect, *R*^2^ = 0.254 and *P = *0.004, weighted UniFrac distance).

**TABLE 1:** Results of differential analyses of bacterial community composition of all the samples using ADONIS and ANOSIM, based on weighted and unweighted UniFrac distances^*a*^

Parameter	Weighted UniFrac				Unweighted UniFrac			
	ADONIS		ANOSIM		ADONIS		ANOSIM	
	*R*²	*P*	*R*	*P*	*R*²	*P*	*R*	*P*
Individual	**0.57**	**0.001**	**0.438**	**0.001**	**0.418**	**0.001**	**0.371**	**0.001**
Sex	0.03	0.257	0.036	0.169	0.025	0.382	0	0.439
NH/DH/SS	0.045	0.538	−0.008	0.564	0.046	0.655	−0.017	0.734
Hands/SS	0.027	0.356	**0.135**	**0.039**	0.023	0.549	−0.01	0.528

a The bold values reprensent significant (*P* < 0.05) variables.

**TABLE 2:** Results of two-way ADONIS analysis of differences in community composition between hands and smartphones, considering individual variation and interaction^*a*^

Parameter	df	SS	*R* ^2^	*F*	*P*
A, individual	13	5.785	**0.572**	4.170	**0.001**
B, hands/SS	1	0.255	**0.027**	2.541	**0.030**
A × B, interaction effects	13	2.657	**0.254**	1.854	**0.001**
Residual	14	1.649	0.148		
Total	41	10.34	1		

a The bold values reprensent significant (*P* < 0.05) variables.

To evaluate the difference in the microbiotas between two hands (DH and NH) regarding their similarity to smartphone microbiotas, Jaccard similarity was calculated for pair comparison in each individual across three sites, including NH, DH, and SS (Fig. 1d and e), which could eliminate the effects of interindividual variation. The similarity between DH and NH was significantly higher than that between SS and NH (*P = *0.003, paired *t* test). Interestingly, the similarity between SS and NH was significantly higher than that between SS and DH (*P = *0.038, paired *t* test). This result indicated that SS microbiotas were more similar to microbiotas from the NH than from the DH, which was validated by the source tracking analysis based on fast expectation-maximization for microbial source tracking (FEAST), which showed significantly more potential sources from the NH than from the DH (*P = *0.008, paired *t* test). Differential analysis revealed that some microbes were significantly enriched or depleted on the SS (Fig. 1f and Fig. S1). For example, *Cutibacterium* and Staphylococcus were significantly depleted on the SS, whereas UCG-002 was enriched on the SS. Consistently, we also found asymmetry between hands compared with the SS at the genus level, e.g., for *Bifidobacterium* (lower on the NH) and *Subdoligranulum* (lower on the DH). Accordingly, these results not only indicated that bacteria are affected by the SS but also suggested the presence of asymmetry of the DH and NH from the microbiological perspective.

With regard to potential pathogens, several opportunistic pathogens, such as Haemophilus parainfluenzae (1.89%), Haemophilus influenzae (0.32%), Corynebacterium durum (0.13%), Corynebacterium tuberculostearicum (3.87%), and Corynebacterium amycolatum (0.13%), were detected on the SS. Additionally, functional profiles were predicted and a potential pathogenic phenotype (predicted by BugBase) and a functional subset related to human disease (predicted by Tax4Fun2) were found on the SS (Fig. S2 and S3), suggesting pathogenic potential on the SS.

### Stronger deterministic processes in smartphone microbiotas.

**FIG 2:**
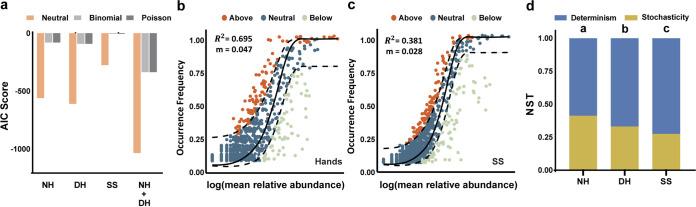
Stronger deterministic processes affect smartphone microbiotas. (a) AIC scores comparing the performances of the neutral, binomial, and Poisson models for explaining the community assembly process. (b and c) Sloan neutral model prediction of microbiota on hands (b) and SS (c), indicated by *R*^2^ values (fit to neutral assembly process) and *m* values (estimated migration rate). Operational taxonomic units (OTUs) are represented by data points and colored according to whether the taxon fit above, within, or below the 95% confidence interval (dashed lines). (d) Taxonomic normalized stochasticity ratio (NST) among the NH, DH, and SS.

Unexpectedly altered phylogenetic diversity (Fig. 1c) and community structure on the SS (Table 2) suggested a disagreement in the assembly processes (e.g., the selection pressure) between the SS and hands. To further elucidate the assembly processes, we introduced Sloan’s neutral model. Model fitness was indicated by lower Akaike information criterion (AIC) scores (Fig. 2a), which showed better performance of the neutral model than binomial and Poisson models in predicting the assembly of smartphone microbiotas. Consistent with the lower phylogenetic diversity, the communities on SSs exhibited a lower *R*^2^ value (0.381 versus 0.695) and migration rate (0.028 versus 0.047) than the communities on hands (Fig. 2b and c). The lower *R*^2^ value indicated a more niche-based (selection; determinism), rather than neutral or stochastic, community assembly processes on the SS, while low migration rates revealed that smartphone microbiotas were limited by dispersal. Normalized stochastic ratios (NST) were calculated and revealed a significantly lower proportion of stochasticity in the SS community (Fig. 2d). Consistent trends were observed using another data set (Fig. S4), confirming that deterministic processes exerted greater influence on the community on the SS.

### Interindividual variation and owner identification.

**FIG 3:**
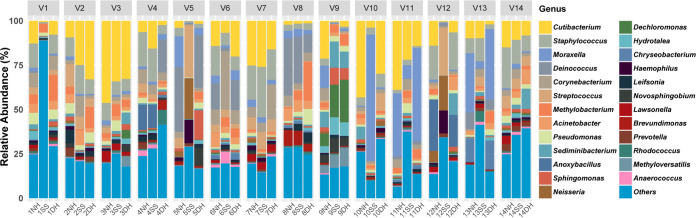
The bacterial taxonomic composition of microbiotas on smartphones and the owners’ hands. The 25 most abundant genera on the NH, DH, and SS of each individual are shown.

**FIG 4:**
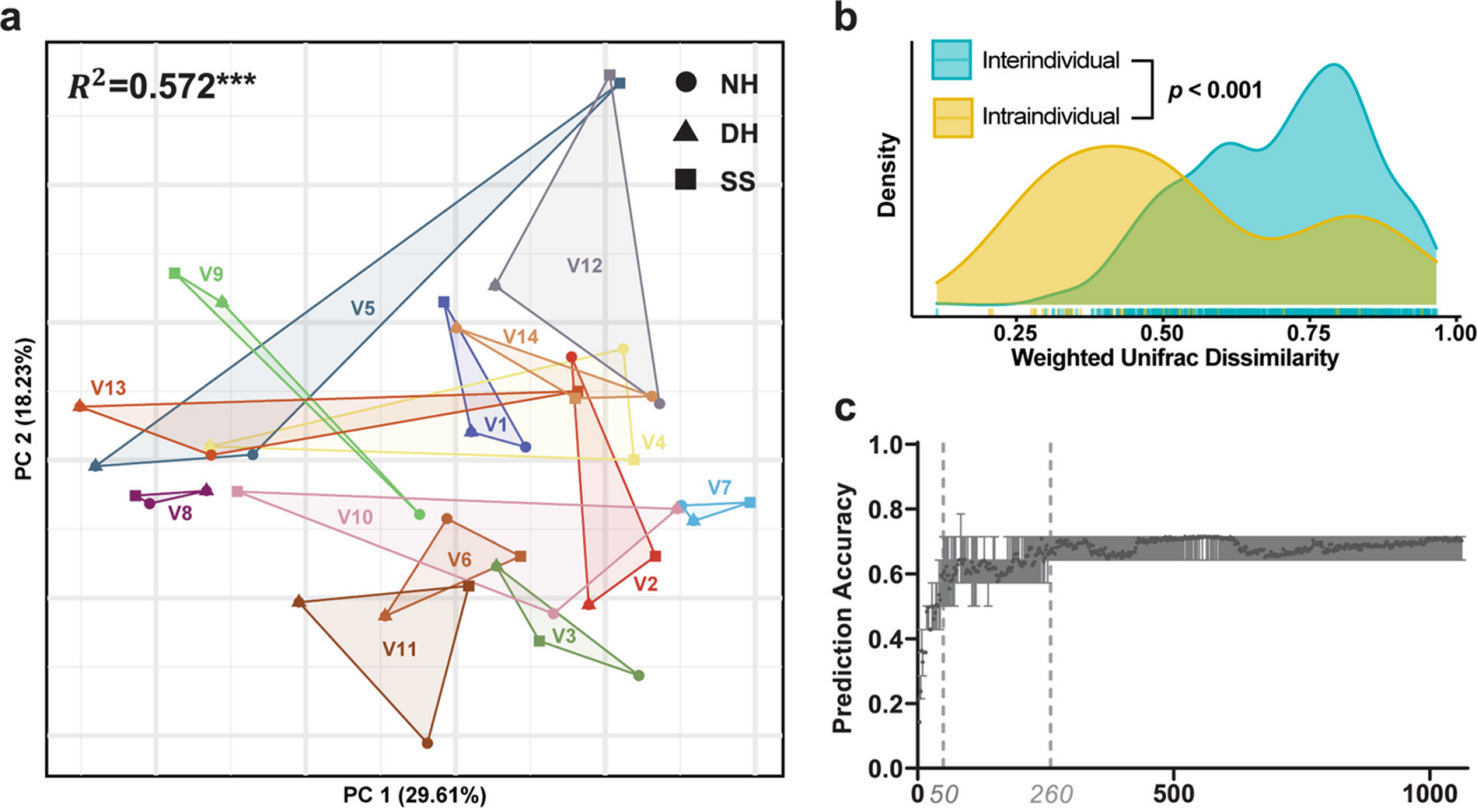
Interindividual variation in the microbial communities on smartphones and hands. (a) Multidimensional scaling (MDS) based on weighted UniFrac distance. Point shapes represent sampling sites. Circles, triangles, and squares indicate samples from the NH, DH, and SS, respectively. Different colors indicate samples from different individuals. The *R*^2^ value represents interindividual variation (PERMANOVA). (b) Distribution of weighted UniFrac distances calculated between samples from different individuals and between samples from the same individual. A Wilcoxon test produced a significant *P* value lower than 0.001, suggesting that samples from the same individual share a large degree of their microbial communities. (c) The prediction accuracy distribution of RF with a given number of OTUs. ***, *P *≤ 0.001.

We further evaluated the intra- and interindividual variation in microbiome composition at the genus level. Most of the bacterial communities consisted of *Cutibacterium* (14.29%), Staphylococcus (9.08%), and *Moraxella* (8.02%) (Fig. 3). We found that the microbiome composition of the hand and SS samples corresponding to each individual were similar. For example, *Cutibacterium* was more abundant in the samples from volunteer no. 3 (V3) than those from the other individuals. In addition, *Deinococcus* was primarily associated with V10. Interindividual variations were also observed in dimension reduction analyses (Fig. 4a and Fig. S5), which was further supported by the distribution of intraindividual and interindividual dissimilarity (Fig. 4b). The abundances of the core OTUs also exhibited intraindividual similarity but interindividual dissimilarity (*P < *0.01, Kruskal-Wallis H test [Fig. S6]).

To assess whether the SS microbiota could serve as a fingerprint of its owner, random forest (RF) classification was performed for owner identification. The hand microbiotas of all 14 individuals were used as training sets, and the SS microbiotas were used as validation sets. We obtained an average prediction accuracy of 70.6%, which is much higher than the accuracy of random expectation (7.14%). In addition, owner identification was conducted with OTU tables generated by different OTU filtering strategies. Only a slight increase in prediction accuracy was observed (Table S6). In contrast to the 97% cluster threshold, we also examined the OTUs clustered at the 99% threshold for identification and obtained only 64% prediction accuracy, implying the presence of noise within OTUs when using a 97% clustering threshold. Finally, to evaluate how many dominant OTUs (at 97% similarity) are enough for owner identification, we calculated the distribution of the prediction accuracy of the RF models with 2 to 1,062 OTUs (Fig. 4c). The prediction accuracy increased rapidly to 60% with the increase in OTUs within the interval from 0 to 50. Then accuracy increased slightly and gradually stabilized with the rise in OTU number from 50 to 260.

### The microbe-microbe correlation and microbial metabolic activity on smartphones.

**FIG 5:**
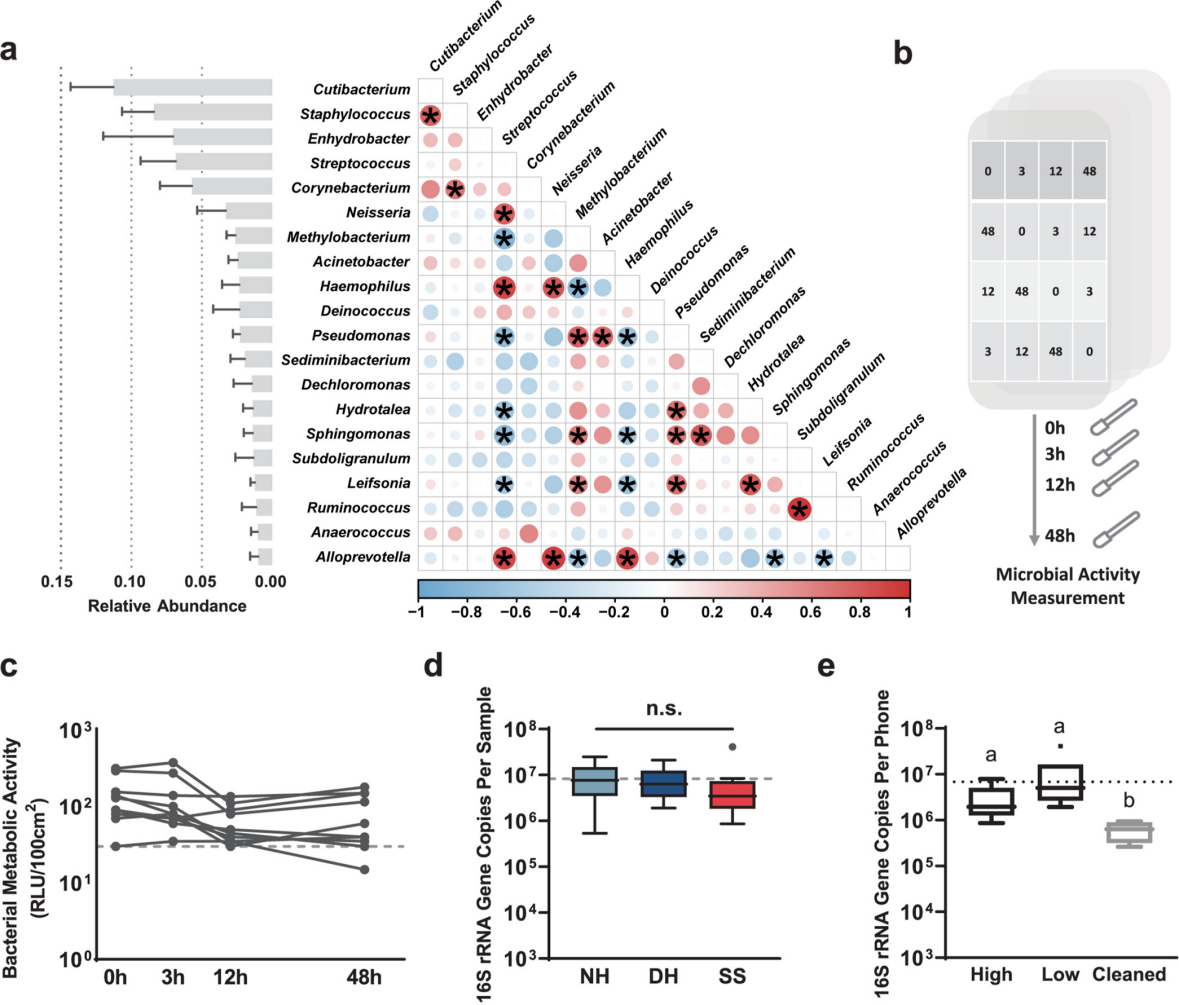
Microbe-microbe correlation, metabolic activity, and bacterial load on the smartphone surface. (a) The left bar plot represents the relative abundance of genera within smartphone samples. The error bar represents the standard error. The right panel shows the pairwise correlation (the proportionality of change [Rho]) matrix for the 20 most abundant genera on smartphones. Significant Rho coefficients were marked with a 0.61 cutoff (a final FDR estimate of 0.05). Circle color and size represent the correlation strength. (b) Schematic representation of the ATP test for measuring microbial metabolic activity on smartphone surfaces at different time points. Subareas with the same number were used to measure activity at a certain time point. (c) Temporal dynamics of bacterial activity on smartphone surfaces at different time points, determined by ATP using a bacterium-specific bioluminescence assay (*n* = 10). RLU, relative light unit. The gray dashed line represents the reference hygiene threshold (30 RLU per 100 cm^2^). (d) Boxplot of 16S rRNA gene copies on NH, DH, and SS (Kruskal-Wallis H test). The dashed line corresponds to the average 16S rRNA gene copies in all the measured samples. Boxes that do not share a letter are significantly different. Dots represent outliers. (e) Boxplot of 16S rRNA gene copies on smartphone touchscreens with different owner cleaning frequencies (high cleaning frequency, daily and weekly; low cleaning frequency, monthly and longer) and with simple cleaning using potable water. The dashed line corresponds to the average 16S rRNA gene copies on smartphone touchscreens without recent cleaning.

To explore the potential interactions among smartphone microbiotas, we performed a correlation test at the genus level (Fig. 5a). The results indicated strong correlations (|Rho| > 0.61 and false-discovery rate [FDR] < 0.05) among some genera, albeit with the high interindividual variation described above. Correlations among community members suggest that there might be microbial activity (not transient or dead bacteria but potentially active) on the SS. Thus, we assessed the temporal dynamics of bacterial activity on the SS by measuring the ATP contents. ATP levels were determined by a bioluminescence assay designed to exclusively measure intracellular bacterial ATP and are displayed as relative light units (RLU). To minimize sampling bias, we sampled four subareas of each SS at each time point (Fig. 5b). The results showed that the bacterial activities (RLU) on the smartphones were detectable for at least 48 h (Fig. 5c). The microbial activity remained stable during the first 3 h (*P = *0.153, Wilcoxon signed-rank test) but decreased within the next 9 h (*P = *0.024, Wilcoxon signed-rank test; the median value decreased by approximately 49.4%). The median RLU values at four time points for each individual ranged from 47.5 to 110 per 100 cm^2^, which exceeded the hygiene threshold given by the manufacturer, namely, 30 RLU per 100 cm^2^ (an alcohol-sterilized smartphone or a new smartphone had values below 10 RLU per 100 cm^2^). The metabolic activity on the SS could represent active microbes and, more importantly, could also partially reflect activity of potential pathogens.

### Enumeration and visualization of bacteria on smartphones.

Given the potential pathogenic bacteria detected on the smartphone, we further assessed the bacterial load by applying absolute qPCR with the universal primer. The average number of 16S rRNA gene copies on hands and the SS were 6.89 × 10^6^ and 8.90 × 10^6^, respectively (Fig. 5d). In addition, the number of bacteria was estimated by the rRNA Operon Copy Number Database (*rrndb*) based on taxonomic composition, which demonstrated an average of 1.75 × 10^6^ bacteria on a smartphone in this study. It is worth noting that the actual microbial counts could be more due to possible incomplete sampling of biomass on the SS.

Moreover, we divided the smartphone samples into two groups, high (daily or weekly) and low (monthly or more) cleaning frequencies based on the results of a questionnaire, and compared their bacterial loads (Fig. 5e). Surprisingly, no significant difference was found (*P = *0.13, Wilcoxon rank sum test), suggesting the rapid recovery of bacterial load due to high contact frequency during daily use. To test the effects of cleaning/hygiene procedures on bacterial reduction, we randomly selected and cleaned smartphones with only tap water and paper napkins and then measured their bacterial loads under the same conditions. Notably, simple SS cleaning/hygiene procedures significantly reduced (*P < *0.01, Wilcoxon rank sum test) the bacterial load, by approximately 1 order of magnitude (Fig. 5e).

**FIG 6:**
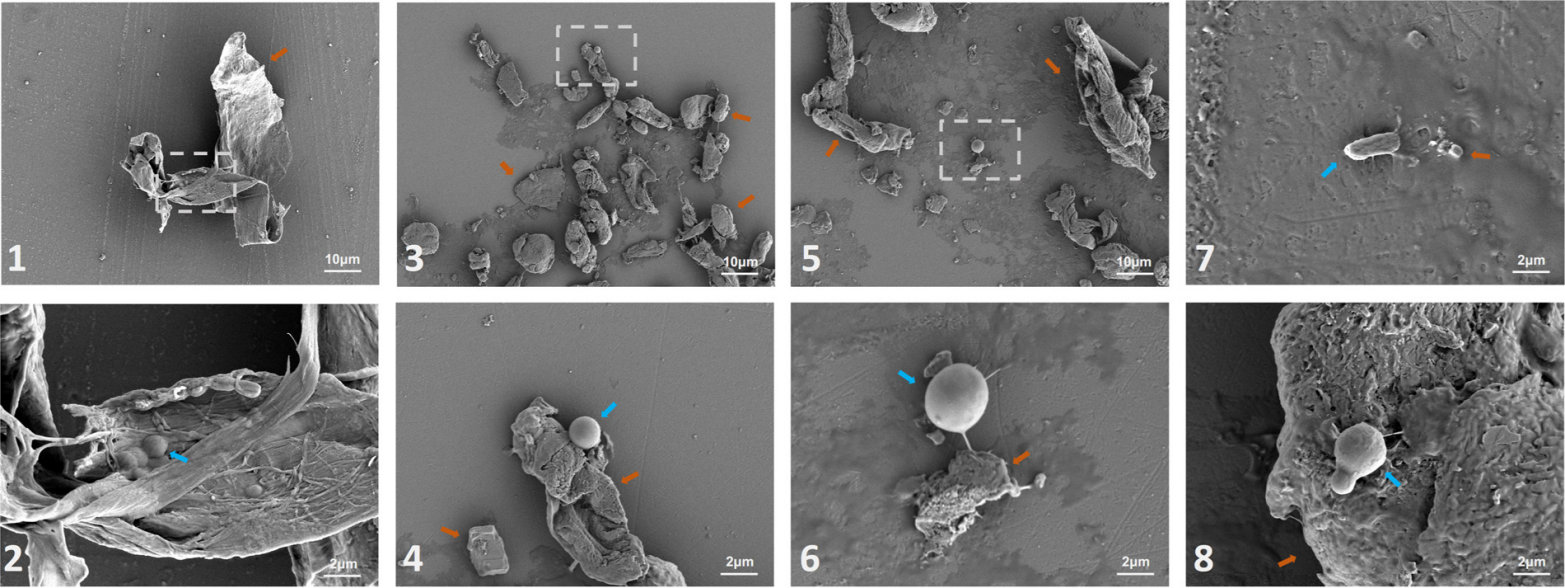
Scanning electron micrographs of the smartphone surface. Images 1, 3, and 5 show debris on the SS magnified by 1,000 times. The zoomed-in images 2, 4, and 6 show a magnified view of the areas highlighted by the white rectangles in images 1, 3, and 5. Images 2, 4, 6, 7, and 8 together showed representative microbes on the SS, magnified 5,000 times. Scale bars are shown on the lower right. (Blue arrow, suspected microbes; orange arrow, certain debris or fragments on the smartphone surface).

Finally, the SS was examined *in situ* using scanning electron microscopy (SEM) (Fig. 6). We found large fragments and various microorganisms, including rod shapes and coccus-like shapes, on the SS.

## DISCUSSION

### Determinism impacts smartphone microbiota assembly.

The principles governing the microbiome on smartphones or other commonly used electronic devices remain poorly understood. Inconsistent with the expectation, our results revealed a deterministic/niche-based assembly process of smartphone microbiotas (to a greater extent than hand microbiotas), as supported by phylogenetic diversity characterization and ecological model analyses. One of the most plausible explanations for this finding is that smartphone microbiotas are not transient during species turnover but likely form a “community” and are affected by determinism. In other words, microorganisms on smartphones can be filtered and selected by environmental factors such as screen material and substrate availability, with fitness differences resulting in the alteration of phylogenetic structures (21). This phenomenon was also reported for microbiotas on microscope oculars (22), suggesting that it is likely a common occurrence among surface microbiomes. Additionally, there were strong correlations among bacteria, albeit with dramatic interindividual differences, thus suggesting the complexity (not only selection pressure on SS but also potential microbe-microbe interactions) underlying the deterministic assembly process of smartphone microbiotas. Consistent with our microbial activity measurement, previous studies found that a considerable proportion of microbiotas on inanimate surfaces (expectedly nonnutritive) are live and stable (23, 24). As suggested by SEM data, commonly touched inanimate surfaces (such as smartphones) are unlikely to be strictly nonnutritive. In contrast, microorganisms could live on the secreta (e.g., sweat and sebaceous gland secretion) and/or other things (e.g., human cell debris and food debris) encountered because of our frequent contact, as evidenced by microenvironments on the skin (25). Collectively, our findings indicate that the smartphone is not only a reservoir of microbes but also a niche in which microbes can survive, be active, and even grow, resulting in decreased phylogenetic diversity and stronger deterministic assembly processes. This has, to our knowledge, never been described before in smartphone microbiota studies. Nonetheless, it should be noted that caution is warranted when inferring processes from DNA-based microecological patterns of small-scale studies. Approaches targeting active microbiota (e.g., metatranscriptomics and culture-based methods) with larger sample sizes might provide a more comprehensive landscape of smartphone microbiota and the principles governing their community assembly.

### Smartphone microbiota features.

By comparing hands, our results demonstrate a previously unknown asymmetry between dominant and nondominant hands from a microbiological point of view. Specifically, dominant and nondominant hands exhibited different microbiome characteristics in certain taxa (such as *Bifidobacterium* and *Lawsonella*) and in their similarity to the smartphone microbiome (the nondominant hand microbiota was more similar than the dominant hand microbiota to the smartphone microbiota). At first glance, it appears paradoxical that the smartphone microbiota is more similar to the microbiota of the nondominant hand. However, the observed hand asymmetry likely originates from genetics, resulting in neural asymmetry and underuse of nondominant hands. Such seemingly contradictory findings might potentially be reconciled by the overuse of the dominant hand, enabling more frequent contact with other objects and the exchange of bacteria, resulting in larger and more stochastic/chaotic fluctuations of the dominant hand microbiota. The difference between dominant and nondominant hands has been described in various studies, such as in rehabilitation science, kinesiology, and neuroscience (26–29). Advanced techniques such as metagenomics may provide novel insights into the microbiological relationship between human hands and smartphones.

Several studies have indicated that microbial community structure could reflect environmental characteristics, such as in corpse decomposition prediction in mammals (30) and cancer diagnostic approaches (31). Given frequent contact with hands, smartphone microbiotas could also reflect characteristics of the owners, enabling us to identify who they are and explore where they may have been recently (15, 32). Concordant with previous research (13), significant interindividual differences between hands and phones were observed, and our results showed an accuracy of identification prediction comparable to that reported by a previous study (33). Based solely on the microbiome composition, some samples were hard to predict, suggesting potential contamination from the environment and the insufficiency of the information recorded for short-read amplicon sequencing-based determination of the microbial community composition (32–34). Therefore, to increase the accuracy of owner identification, higher taxonomic resolution for profiling community composition, together with other characterization techniques (e.g., bacterial pangenome presence/absence), is needed (14). For instance, recent research reported that a combination of microbiota structures and exact sequence variants of Cutibacterium acnes 16S rRNA genes could increase accuracy rates from 71.7% to 93.3% (33).

### Smartphone hygiene.

Pathogen transmission mediated by smartphones has been suggested to be a potential global public health risk (10). Numerous bacteria detected in this study have been reported to be opportunistic pathogens, such as *H. parainfluenzae*, H. influenzae, *C. tuberculostearicum*, and *C. durum* (35–38). Under immunocompromised conditions, opportunistically pathogenic microbiotas can cause severe disease (25). Moreover, some species are naturally competent to exchange genetic material (e.g., antibiotic resistance genes [ARGs]), which could occur on the smartphone surface (39, 40). Previous studies have reported diverse pathogens and ARGs on various inanimate surfaces, e.g., doorknobs (12), hospital devices (23), and paper money (41) and surfaces in metro systems (42). Thus, the contaminants and pathogens on such frequently and regularly touched surfaces may pose a risk of horizontal gene transfer, human infection, and even mortality (42–44).

With regard to the bacterial load on smartphones, previous studies were mostly focused on identifying finite culturable pathogen groups and antibiotic resistance capacity in hospitals (16, 45). In this study, culture-independent qPCR showed a considerably high bacterial load (estimated at 10^6^) on the smartphone touchscreens of healthy university students; the load is much higher than that reported for secondary school students (approximately 10^3^) (46), likely due to the elevated frequency of usage or the different habits of university students. Furthermore, the bioluminescence assay quantitatively demonstrated the ability of microorganisms on smartphones to maintain activity for at least 48 h, consistent with the findings of a previous report based on culture-dependent methods and bacterial viability staining (24, 47). Therefore, smartphones should also be recognized as a potential vector for transmitting pathogens. Smartphones are portable devices that can exert extensive effects on the environment and humans. For example, standardized hand washing/hygiene practices can efficiently reduce or even kill foreign bacteria from the environment, thus restoring the hand microbiota to a natural skin microbiome and decreasing the risk of pathogens spreading to other body parts by the hands (11). Nonetheless, frequent contact with smartphones could undermine hand hygiene efforts since hands can reacquire contaminants and perhaps pathogenic microbiotas from the smartphone reservoir of microbes. In addition to indirect transmission by hands, smartphone surfaces are considered particularly high-risk surfaces since they can come into direct contact with the face or mouth while a person is talking into a smartphone (48). The recolonization of microorganisms might thus exert adverse effects on the user and pose a biothreat risk for infections with the potential to spread globally. Moreover, our results showed a significant reduction in bacterial load even with simple cleaning practices (e.g., with water), which is consistent with the findings of previous studies (19). Although the use of alcohol might damage a phone’s screen, both Apple and Samsung revised their support guidelines during the COVID-19 pandemic (e.g., to include gently wiping the surface using 70% isopropyl alcohol), given that smartphones could be a vector for pathogens. Collectively, these findings emphasize the necessity of smartphone hygiene, including daily cleaning, even simply wiping with water, to reduce potential biothreats. Clarifying the underlying mechanisms responsible for smartphone microbiota assembly is of particular importance under global change. The distinctive features and potential pathogenicity of microorganisms, their considerable loads on smartphones or other surfaces, and particularly their role in pathogen dissemination in our daily lives merit future investigation (47).

### Conclusion.

This study investigated the structure, assembly, quantity, and dynamic metabolic activity of the microbiome on the smartphone surface and its relationship with the microbiome of the owner’s hands using multiple culture-independent techniques. Our results not only indicate that the smartphone could be a niche for microbial activity with deterministic control on community assembly but also demonstrate the health risk of the smartphone microbiota due to the considerable microbial count, stable activity, and the presence of opportunistic pathogens. Comprehensive research on the microbiotas of electronic devices used daily, especially mobile communication devices such as smartphones, could facilitate the development of device cleaning guidelines and strategies to promote public health, particularly under pandemic conditions.

## MATERIALS AND METHODS

### Sample collection.

Samples were collected from the left and right hands of 14 healthy volunteers and the surfaces of their smartphones (i.e., the touchscreen). All the subjects were required not to wash their hands for 2 h before sample collection to ensure bacterial community characterization and biomass collection (49). Subjects were instructed to collect samples using sterilized swabs premoistened with saline for 30 s. Swabs were used immediately for DNA extraction. A questionnaire was administered to the subjects about their sex, dominant hand, whether their phones were film protected, cleaning habits (e.g., wiping the touchscreen using moistened tissue paper or more effective methods), the frequency with which they cleaned their smartphone (high cleaning frequency, daily and weekly; low cleaning frequency, monthly and longer), and whether the cleaning involved sterilization (using the disinfectants such as ethanol, isopropyl alcohol, or Clorox disinfecting wipes). All screen protectors are made of tempered glass, which has properties similar to those of the smartphone surface. To test the bacterial reduction efficiency of simple cleaning/hygiene, we cleaned a smartphone surface by spraying 1 mL of tap water (nonsterile) and wiping it evenly with normal paper napkins (nonsterile) until there were no visible traces of dirt. The samples were handled in a completely anonymous manner and assigned serial numbers from V1 to V14.

### DNA extraction and high-throughput sequencing.

DNA was extracted from 42 samples using a Mag-Bind bacterial DNA 96 kit (Omega, GA, USA) according to the manufacturer’s instructions. The final DNA concentration and purity were measured by a NanoDrop 2000 UV-visible (UV-Vis) spectrophotometer (Thermo Scientific, MA, USA). DNA quality was checked by 1% agarose gel electrophoresis. For high-throughput sequencing, a primer pair (338F-806R) (Table S7) targeting the V3-V4 region was used to generate amplicons (50, 51). The PCRs were conducted using the following program: 3 min of initial denaturation at 95°C; 30 cycles of 30 s at 95°C, 30 s of annealing at 55°C, and 45 s of elongation at 72°C; and a final extension at 72°C for 10 min. PCRs were conducted in triplicate 20-μL mixtures containing 4 μL of 5× FastPfu buffer, 2 μL of 2.5 mM deoxynucleoside triphosphates (dNTPs), 0.8 μL of each primer (5 μM), 0.4 μL of FastPfu polymerase, and 10 ng of template DNA. PCR products were separated on a 2% agarose gel, and target bands were purified using the AxyPrep DNA gel extraction kit (Axygen Biosciences, CA, USA) and then quantified using QuantiFluor-ST (Promega, WI, USA) according to the manufacturer’s protocol. Afterward, purified amplicons were pooled in equimolar concentrations and paired-end sequenced (PE300) on an Illumina MiSeq platform (Illumina, CA, USA) following standard protocols provided by Majorbio Bio-Pharm Technology Co. Ltd. (Shanghai, China). Saline-premoistened swabs exposed to the air for 30 s were processed with the same DNA extraction and PCR amplification kits as negative controls to test for reagent and laboratory contamination (52).

### Data processing.

Raw sequence reads were demultiplexed, quality filtered, and merged as previously described (53, 54). Chimeric sequences were recognized and removed using UCHIME. Operational taxonomic units (OTUs) were clustered using UPARSE (version 7.0 [http://drive5.com/uparse/]) at a 97% similarity cutoff (55). After quality control, we obtained 2,135,282 clean reads in all samples ranging from 9,470 to 71,808, with an average read count of 50,209 and an average length of 419 bp. The taxonomy of each 16S rRNA gene sequence was assigned using the Ribosomal Database Project (RDP) classifier against the Silva (SSU138) 16S rRNA database at a confidence threshold of 0.7. OTUs representing chloroplasts or mitochondria and those with low frequency (<0.05% in all samples) were removed prior to further analyses. Samples were rarefied to a sequencing depth of 9,000 sequences per sample. To ensure that the samples had sufficient read numbers and were paired within each individual, only biological samples with sufficient sequences (more than 9,000) among the hand and smartphone samples were used. For comparison, two extra filtering strategies (keeping OTUs containing reads ≥4 and no filter) were employed, with outputs of 2,385 and 2,805 remaining OTUs, respectively. Rarefaction curves were calculated to ensure that the sequencing depth was sufficient.

### Bacterial quantification.

Absolute quantification of total bacterial 16S rRNA gene copies was conducted by RT-qPCR assay using the Roche LightCycler 480 system (Roche, Basel, Switzerland) as previously described (56). The amplification reaction was performed in triplicate with SYBR green (Vazyme Biotech, Nanjing, China), using a universal primer pair (785F-907R) (Table S7) (57). The thermocycler started with a DNA denaturation step at 95°C for 5 min, followed by 40 cycles of denaturation at 95°C for 10 s, annealing at 60°C for 10 s, and extension at 72°C for 10 s. After amplification, a melting-curve step (from 65°C to 95°C, 0.11°C/s) was performed. Standards were developed from PCR products of the 16S rRNA gene (27F-1492R) (Table S7) from the mixed DNAs from each sample with an equivalent amount and 10-fold serial dilutions thereof. For this, PCR amplicons were purified using an E.Z.N.A. gel extraction kit (Omega, GA, USA), and the concentration was determined using a NanoDrop (Thermo Scientific, MA, USA). A standard curve was made with a series of 10-fold dilutions with the same reaction parameters. The 16S rRNA gene copy numbers for each reaction were calculated from the standard curve. The melting curve was obtained to confirm the appropriate size of the amplified products and remove the undetectable samples from the RT-qPCR analyses. The average copy number of 16S rRNA genes per bacterium is currently estimated at 5.09 based on the rRNA Operon Copy Number Database (58).

### Bacterium-specific bioluminescence assay.

A bacterial metabolic activity assay was performed by determining ATP content on the smartphone surface at four time points by the ATP bioluminescence system (SystemSure Plus; Hygiena, CA, USA), which could break the cell, combine the ATP, and measure the relative light units (RLU) of collection of a single swab. To selectively determine the bacterial ATP (by eliminating human sources), we introduced the following bacterium-specific bioluminescence assay modified from references 59 and 60. First, after biomass collection, the swab was mixed with 90 μL of Triton X-100 (Solarbio, Beijing, China) for 2 min to lyse somatic cells; 0.1 U of apyrase and 10 μL of 10× reaction buffer (New England Biolabs, Ipswich, England) were added to catalyze the hydrolysis of ATP from the outer bacterial cell membranes (e.g., human cells) over a period of 10 min at 30°C, followed by 20 min at 65°C to inactivate the apyrase. Finally, RLUs were measured with an ATP bioluminescence system according to the manufacturer’s instructions (SystemSure Plus; Hygiena, CA, USA). To track the microbial activity over a 48-h period, 10 additional smartphones that had not been sterilized in 24 h were collected. To minimize the sampling bias, we delineated a 6.5-cm by 12-cm area and divided it into 16 subareas with the same size. Each swab was utilized to sample four subareas (approximately 20 cm^2^). ATP hygiene monitoring thresholds were established according to the instruction manual. Notably, the actual ATP content could be greater than that detected due to the possible incomplete collection by swabs.

### SEM.

Difference analysis of bacterial community structure and bacterial load showed no significant differences between film-protected and non-film-protected smartphones, suggesting no obvious influence of screen protective films on microbes on the contacted smartphone surface. Therefore, we employed smartphone protector films as an alternative for scanning electron microscopy (SEM) observation. Smartphone protector films were cut into 1-cm^2^ subareas as SEM specimens. Specimens were fixed in 2.5% glutaraldehyde in phosphate buffer (0.1 M, pH 7.0) for more than 4 h and then rinsed three times in phosphate buffer (0.1 M, pH 7.0) (15 min each time). Then specimens were postfixed with 1% osmium tetroxide (OsO_4_) in phosphate buffer (0.1 M, pH 7.0) in a fume hood for 1 to 2 h at room temperature and washed three times in phosphate buffer (0.1 M, pH 7.0) for 15 min at each step. Afterward, specimens were dehydrated in a series of ethanol solutions (50%, 70%, 80%, 90%, 95%, and 100%) for 20 min at each step and incubated with absolute ethanol for 20 min, followed by dehydration in a Hitachi model HCP-2 critical point dryer. The dehydrated sample was coated with gold-palladium in a Hitachi model E-1010 ion sputter for 4 to 5 min and examined using a GeminiSEM 300 field emission scanning electron microscope (Zeiss, Göttingen, Germany).

### Statistical analyses.

All statistical analyses were performed in the R environment (v3.6.3). Normal distribution and homoscedasticity were assessed by Shapiro-Wilk and Levene’s tests, respectively. The Chao1 index and Faith’s phylogenetic diversity (PD) index were calculated using the R packages “vegan” and “picante.” Microbiota dissimilarity between samples was represented by dimension reduction analysis, using Jaccard and Bray-Curtis distances (taxonomic composition) and unweighted and weighted UniFrac distances (phylogenetic composition) as well as Aitchison distance (taxonomic composition) and PhILR Euclidian distance (phylogenetic composition) (61, 62). The analyses of variation in bacterial community structure between different groups were performed by permutational multivariate analysis of variance (PERMANOVA) with the adonis function and analysis of similarities (ANOSIM) with anosim function, using the R package vegan. Source track analysis was performed by fast expectation-maximization for microbial source tracking (FEAST) using hand microbiota from each individual as the “source” and their smartphone microbiota as the “sink” as previously described (63, 64). Differential analysis was performed using DESeq2 and analysis of compositions of microbiomes with bias correction (ANCOM-BC) (65, 66). Functional prediction was performed using the R package “Tax4Fun2” (67). Organism-level microbiome phenotypes were predicted using BugBase (https://bugbase.cs.umn.edu/). To evaluate the degree of neutral or niche-based community assembly process, Sloan’s neutral model (68) analysis was performed based on species abundance distribution, as previously described (69, 70). Briefly, the microbial community group with a higher *R*^2^ value is consistent with the neutral bacterial community assembly process. The estimated migration rate (*m*) represents the dispersal limitation. The neutral model fit was compared with the binomial and Poisson models based on Akaike information criterion (AIC) scores. The taxonomic normalized stochasticity ratio (NST) was used to measure the importance of the stochastic ratio in the process of community assembly (ranging from 0 to 1) (71), that is, whether the community assembly was dominated by deterministic (<50%) or stochastic (>50%) processes.

For owner identification, a random forest method was implemented with the “randomForest” package in R (72). In each test, 6,000 decision trees were generated. The hand samples were set as the training set, and smartphone samples were set as the validation set with 100 repeats. The model accuracy was calculated from the out-of-bag (OOB) error rate. Owner identification was determined with two other OTU tables produced by different filtering strategies. To evaluate how many of the most abundant OTUs were needed for owner identification in this study, we calculated the distribution of prediction accuracy for a given number within 2 to 1,062 based on RF modeling. RF modeling was conducted at a given number of OTUs. The 20 most abundant genera detected in the smartphone samples were utilized for correlation analysis. For each genus pair, we measured proportionality of change, i.e., Rho metrics, a rigorous method for calculating the correlations in compositional microbiome data, using the R package “propr” (73, 74). The *P* values from multiple tests were corrected with false-discovery rate (FDR) using the “p.adjust” function in R.

### Data availability.

The raw sequencing data have been deposited into the NCBI Sequence Read Archive (SRA) database under accession number PRJNA657188.
