# Supplementary material for: Smartphones as an Ecological Niche of Microorganisms: Microbial Activities, Assembly, and Opportunistic Pathogens
Source: Microbiol Spectr. 2022 Aug 30;10(5):e01508-22. doi: 10.1128/spectrum.01508-22 (PMC9603676; doi:10.1128/spectrum.01508-22)
Supplement: Supplemental file 1 — Supplemental material. Download spectrum.01508-22-s0001.pdf, PDF file, 0.5 MB [file spectrum.01508-22-s0001.pdf]

Supplementary tables

Table S1 Detailed information of sampling subjects.

Person	Sex	Phone Film	Clean Frequency	Clean Frequency
V1	Female	Film	Less than monthly	Daily
V2	Female	Film	More than monthly	Longer than Monthly
V3	Female	No Film	More than monthly	Only Dirty
V4	Female	Film	More than monthly	Monthly
V5	Male	Film	More than monthly	Longer than Monthly
V6	Female	Film	Less than monthly	Weekly
V7	Male	Film	Less than monthly	Daily
V8	Male	Film	Less than monthly	Daily
V9	Female	No Film	More than monthly	Longer than Monthly
V10	Female	Film	Less than monthly	Daily
V11	Male	Film	Less than monthly	Weekly
V12	Male	Film	More than monthly	Longer than Monthly
V13	Female	Film	More than monthly	Longer than Monthly
V14	Male	No Film	More than monthly	Longer than Monthly

Table S2 The average relative abundances and five taxonomy levels of the core OTUs.

OTU number	Relative Abundance	Phylum	Order	Family	Genus	Species
OTU1024	13.89%	Actinobacteriota	Propionibacteriales	Propionibacteriaceae	*Cutibacterium*	*Cutibacterium acnes*
OTU1279	8.84%	Firmicutes	Staphylococcales	Staphylococcaceae	*Staphylococcus*	*unclassified*
OTU827	8.02%	Proteobacteria	Pseudomonadales	Moraxellaceae	*Moraxella*	*Moraxella osloensis*
OTU590	3.61%	Firmicutes	Lactobacillales	Streptococcaceae	*Streptococcus*	*unclassified*
OTU1611	3.26%	Proteobacteria	Rhizobiales	Beijerinckiaceae	*Methylobacterium*	*Methylobacterium brachiatum*
OTU375	3.05%	Actinobacteriota	Corynebacteriales	Corynebacteriaceae	*Corynebacterium*	*Corynebacterium tuberculostearicum*
OTU270	1.60%	Proteobacteria	Pseudomonadales	Moraxellaceae	*Acinetobacter*	*Acinetobacter johnsonii*
OTU2738	1.25%	Actinobacteriota	Micrococcales	Microbacteriaceae	*Leifsonia*	*Leifsonia shinshuensis*
OTU1819	1.13%	Proteobacteria	Sphingomonadales	Sphingomonadaceae	*Sphingomonas*	*unclassified*
OTU1547	0.82%	Actinobacteriota	Corynebacteriales	Corynebacteriaceae	*Corynebacterium*	*unclassified*
OTU1222	0.77%	Actinobacteriota	Corynebacteriales	Nocardiaceae	*Rhodococcus*	*Rhodococcus erythropolis*
OTU2100	0.57%	Bacteroidota	Chitinophagales	Chitinophagaceae	*Sediminibacterium*	*uncultured*
OTU440	0.56%	Proteobacteria	Burkholderiales	Comamonadaceae	*Delftia*	*Delftia tsuruhatensis*
OTU450	0.53%	Actinobacteriota	Micrococcales	Micrococcaceae	*Micrococcus*	*Micrococcus luteus*
OTU1945	0.51%	Proteobacteria	Rhizobiales	Xanthobacteraceae	*Bradyrhizobium*	*Bradyrhizobium japonicum*
OTU2344	0.15%	Proteobacteria	Aeromonadales	Aeromonadaceae	*Aeromonas*	*Aeromonas caviae*

Table S3 The alpha diversities of microbial communities on hands and smartphone surface with the p values representing the comparison of the alpha diversity index between hands and smartphone group.

	Richness	Chao1	Shannon	Faith's PD
Hands (L,R)	225.46±8.07	257.76±8.87	3.35±0.10	20.05±0.92
Smartphone (S)	197.5±14.67	226.78±19.52	3.2±0.17	17.19±0.95
P value (t test)	0.11	0.165	0.457	**0.038**

Table S4 Differential analyses of bacterial community composition of all the samples from different sites, subjects, and sexes, with two statistical tests, ADONIS and ANOSIM, based on Bray–Curtis and Jaccard distance.

	Bray-Curtis				Jaccard			
	ADONIS		ANOSIM		ADONIS		ANOSIM	
	R^2	*P*	*R*	*P*	*R^2*	*P*	*R*	*P*
Individual	0.558	**0.001**	0.473	**0.001**	0.430	**0.001**	0.584	**0.001**
Sex	0.032	0.176	0.030	0.191	0.028	0.112	0.024	0.253
Position(L/M/R)	0.043	0.663	0	0.574	0.044	0.892	0	0.930
Hand/Phone	0.024	0.449	0.136	**0.046**	0.023	0.605	0.007	0.426

Table S5 Differential analyses of bacterial community composition of smartphone samples.

	Bray-Curtis				Weighted Unifrac				Jaccard				Unweighted Unifrac			
	ADONIS		ANOSIM		ADONIS		ANOSIM		ADONIS		ANOSIM		ADONIS		ANOSIM	
	R^2	P	R	P	R^2	P	R	P	R^2	P	R	P	R^2	P	R	P
Sex	0.066	0.720	0.000	0.654	0.053	0.917	0.000	0.818	0.066	0.720	0.000	0.654	0.053	0.917	0.000	0.818
PhoneFilm	0.072	0.538	0.017	0.425	0.077	0.435	0.025	0.454	0.072	0.538	0.017	0.425	0.077	0.435	0.025	0.454
Clean Frequency	0.083	0.331	0.001	0.426	0.089	0.254	0.000	0.588	0.083	0.331	0.001	0.426	0.089	0.254	0.000	0.588
Sterilization	0.079	0.369	0.088	0.296	0.074	0.521	0.000	0.875	0.079	0.369	0.088	0.296	0.074	0.521	0.000	0.875

Table S6 Three different OTU filtering strategies with similar owner identification accuracy by random forest (RF).

Filter Strategy	Remaining OTU	Prediction Accuracy
Strategy 1	1062	70.60%
Strategy 2	2385	71.40%
Strategy 3	2805	71.40%

Table S7 Primers used in this study.

Primers	Sequence	Usd for	Reference
338F	5’-ACTCCTACGGGAGGCAGCAG-3’	Amplicon sequencing	Chen et al., 2018
806R	5’- GGACTACHVGGGTWTCTAAT-3’		
785F	5’-GGATTAGATACCCTGGTA-3’	qPCR	Hülsdünker et al., 2018
907R	5’-CCGTCAATTCCTTTRAGTTT-3’		
27F	5′-AGAGTTTGATCMTGGCTCAG-3′	qPCR	Chen et al., 2018
1492R	5′-TACGGYTACCTTGTTACGACTT-3′		

Hulsdunker J, Ottmuller KJ, Neeff HP, Koyama M, Gao Z, Thomas OS, Follo M, Al-Ahmad A, Prinz G, Duquesne S, Dierbach H, Kirschnek S, Lammermann T, Blaser MJ, Fife BT, Blazar BR, Beilhack A, Hill GR, Hacker G, Zeiser R. 2018. Neutrophils provide cellular communication between ileum and mesenteric lymph nodes at graft-versus-host disease onset. Blood 131:1858-1869. https://doi.org/10.1182/blood-2017-10-812891

Chen B, Du K, Sun C, Vimalanathan A, Liang X, Li Y, Wang B, Lu X, Li L, Shao Y. 2018. Gut bacterial and fungal communities of the domesticated silkworm (Bombyx mori) and wild mulberry-feeding relatives. ISME J 12:2252-2262. https://doi.org/10.1038/s41396-018-0174-1

Supplementary figures

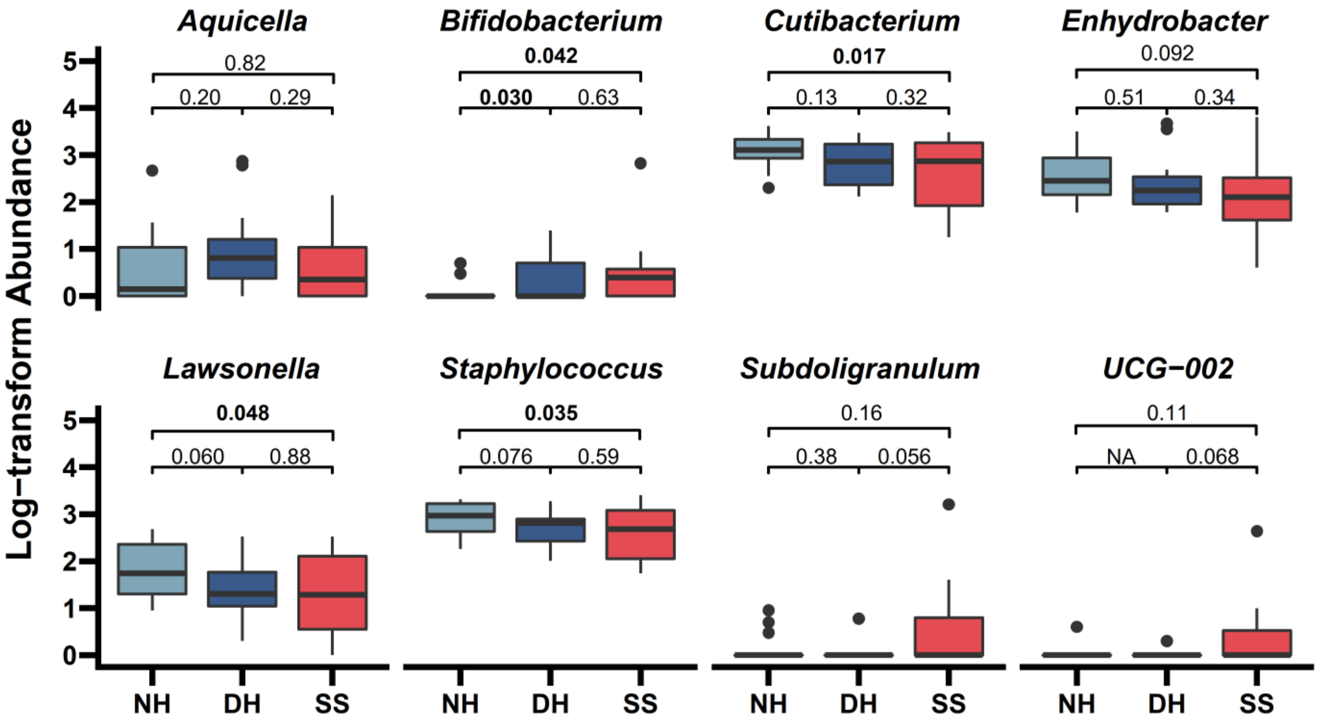

Fig. S1 Abundances of significantly changed genera in each group based on ANCOM-BC.

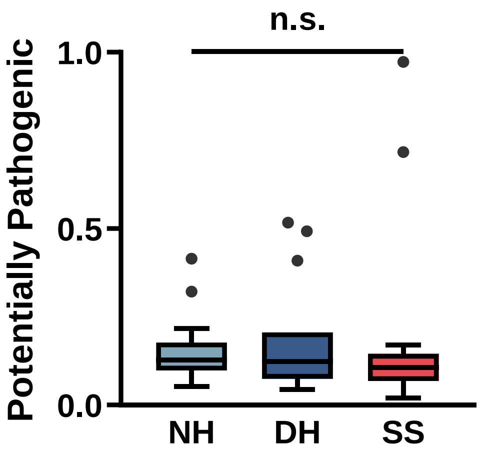

Fig. S2 Boxplot of the prediction of the potential pathogenic phenotype of bacteria on NH, DH and SS (predicted by BugBase).

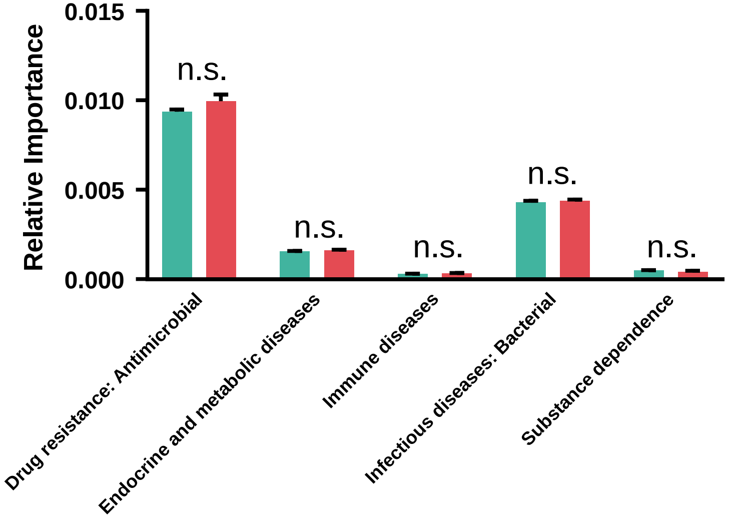

Fig. S3 Bar plot displaying the functional prediction of the bacterial community on hands and smartphone surface at the level 2 pathway profile related to human disease (n.s. = $p > 0.05$, t-test).

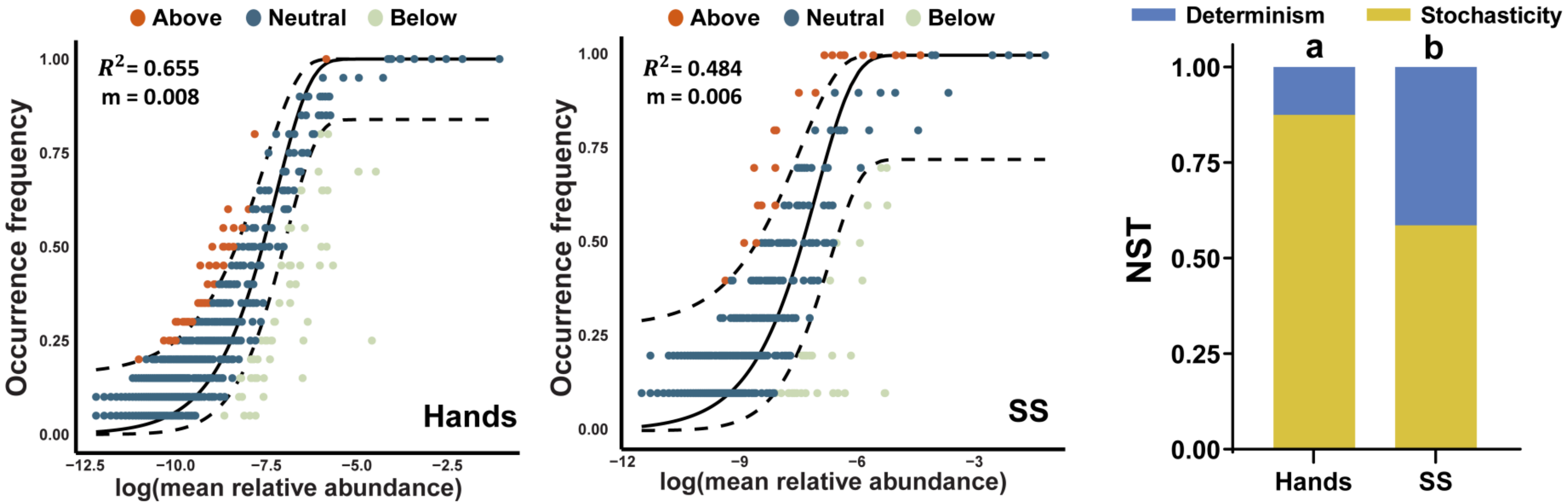

Fig. S4 Sloan neutral model and normalized stochastic ratio (NST) analysis using an independent dataset (DRA008105).

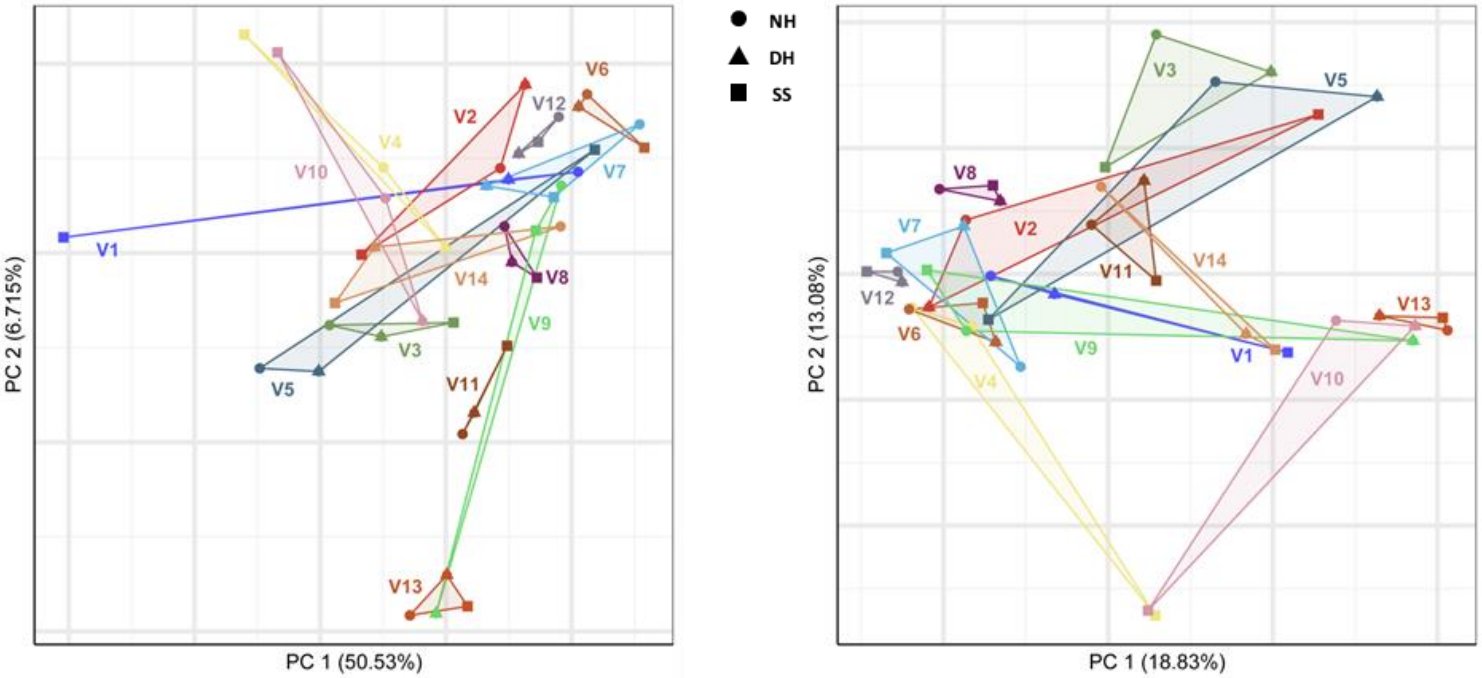

Fig. S5 Dimension reduction in microbiome data using PhILR-based PCoA and Aitchison distance-based PCA.

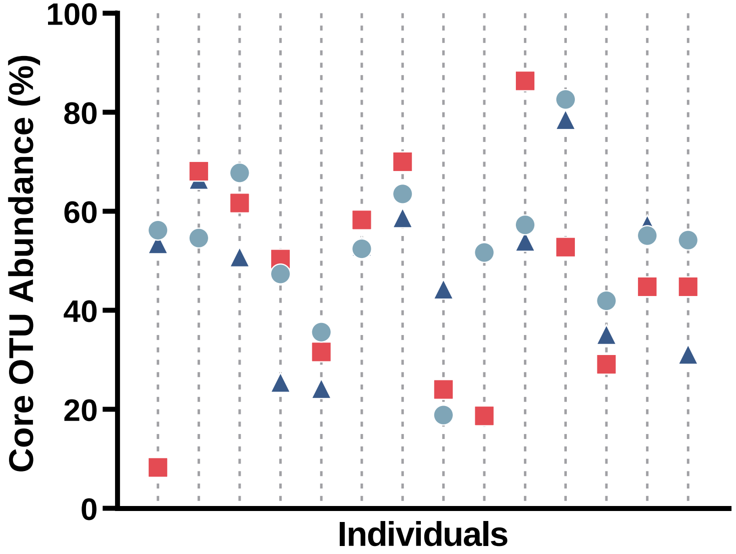

Fig. S6 The relative abundances of all the core OTUs in the surveyed 14 individuals, indicated by the dotted dashes.
